# Poliovirus Vaccination Induces a Humoral Immune Response That Cross Reacts With SARS-CoV-2

**DOI:** 10.3389/fmed.2021.710010

**Published:** 2021-08-03

**Authors:** Brittany A. Comunale, Lilly Engineer, Yong Jiang, John C. Andrews, Qianna Liu, Lyuqing Ji, James T. Yurkovich, Roderick A. Comunale, Qiyi Xie

**Affiliations:** ^1^Johns Hopkins Bloomberg School of Public Health, Department of Health Policy and Management, Johns Hopkins University, Baltimore, MD, United States; ^2^Johns Hopkins School of Medicine, Department of Anesthesiology and Critical Care Medicine, Baltimore, MD, United States; ^3^America Diagnostics, San Diego, CA, United States; ^4^E-Mo Biology, Inc., Arcadia, CA, United States; ^5^Aivance Health, Inc., Jacksonville, FL, United States

**Keywords:** SARS-CoV-2, adaptive immunity, poliovirus, immunization, cross-reactivity, RdRp protein

## Abstract

**Background:** Millions have been exposed to SARS-CoV-2, but the severity of resultant infections has varied among adults and children, with adults presenting more serious symptomatic cases. Children may possess an immunity that adults lack, possibly from childhood vaccinations. This retrospective study suggests immunization against the poliovirus may provide an immunity to SARS-CoV-2.

**Methods:** Publicly available data were analyzed for possible correlations between national median ages and epidemiological outbreak patterns across 100 countries. Sera from 204 adults and children, who were immunized with the poliovirus vaccine, were analyzed using an enzyme-linked immunosorbent assay. The effects of polio-immune serum on SARS-CoV-2-induced cytopathology in cell culture were then evaluated.

**Results:** Analyses of median population age demonstrated a positive correlation between median age and SARS-CoV-2 prevalence and death rates. Countries with effective poliovirus immunization protocols and younger populations have fewer and less pathogenic cases of COVID-19. Antibodies to poliovirus and SARS-CoV-2 were found in pediatric sera and in sera from adults recently immunized with polio. Sera from polio-immunized individuals inhibited SARS-CoV-2 infection of Vero cell cultures. These results suggest the anti-D3-pol-antibody, induced by poliovirus vaccination, may provide a similar degree of protection from SARS-CoV-2 to adults as to children.

**Conclusions:** Poliovirus vaccination induces an adaptive humoral immune response. Antibodies created by poliovirus vaccination bind the RNA-dependent RNA polymerase (RdRp) protein of both poliovirus and SARS-CoV-2, thereby preventing SARS-CoV-2 infection. These findings suggest proteins other than “spike” proteins may be suitable targets for immunity and vaccine development.

## Introduction

Older adults have an increased likelihood of experiencing more severe COVID-19, compared to children under 18 (1–3). Such age-dependent associations were observed during the 2003 SARS-CoV epidemic, as older adults were more likely to contract or die from the disease, compared to younger individuals (4). This apparent immunity to SARS-CoV and SARS-CoV-2 infection may be associated with childhood vaccinations, such as the poliovirus vaccine. While the poliovirus vaccine has been administered to 90% of the world's population, antibodies induced by the poliovirus vaccine diminish over time, almost completely by young adulthood (5).

Early clinical studies have shown that some vaccines, including the poliovirus vaccine, can not only protect individuals from the virus for which it was created (polio), but also from other, structurally related viruses (6). A recent analysis has demonstrated an inverse correlation between susceptibility to SARS-CoV-2 and the severity of COVID-19, and the titers of mumps antibodies (7).

SARS-CoV-2 and the poliovirus are positive ssRNA viruses, whose genomic RNA can be directly used as a protein translation template, as well as for genomic replication driven by an RNA-dependent RNA polymerase (RdRp) protein that is translated from the viral template (8, 9). Due to the importance of the RdRp protein in viral replication, it is the primary target for anti-virus drug screening (10). Thus, the structural similarities in the RdRp of all single-stranded, positive sense RNA viruses may explain the cross-reactivity of polio-immune serum with SARS-CoV-2 antigens (11).

This retrospective study shows that similarities between poliovirus RdRp and SARS-CoV-2 RdRp may explain the apparent protection against SARS-CoV-2 provided by poliovirus vaccination. Two poliovirus vaccine formulations have been used in the worldwide campaign to eradicate polio: an oral live poliovirus vaccine (OPV), and an inactivated poliovirus vaccine (IPV) (12). OPV is no longer administered in the United States over concerns for the spread of vaccine-derived-poliovirus (VDPV), as there is a remote possibility of VDPV escape into the environment with the live vaccine. IPV, on the other hand, is the form of poliovirus vaccination employed in the United States, due to its zero risk of VDPV, strong efficacy, and minimal risk of side effects. Thus, the study presented here evaluates sera from individuals that were immunized with IPV.

**Figure 1:**
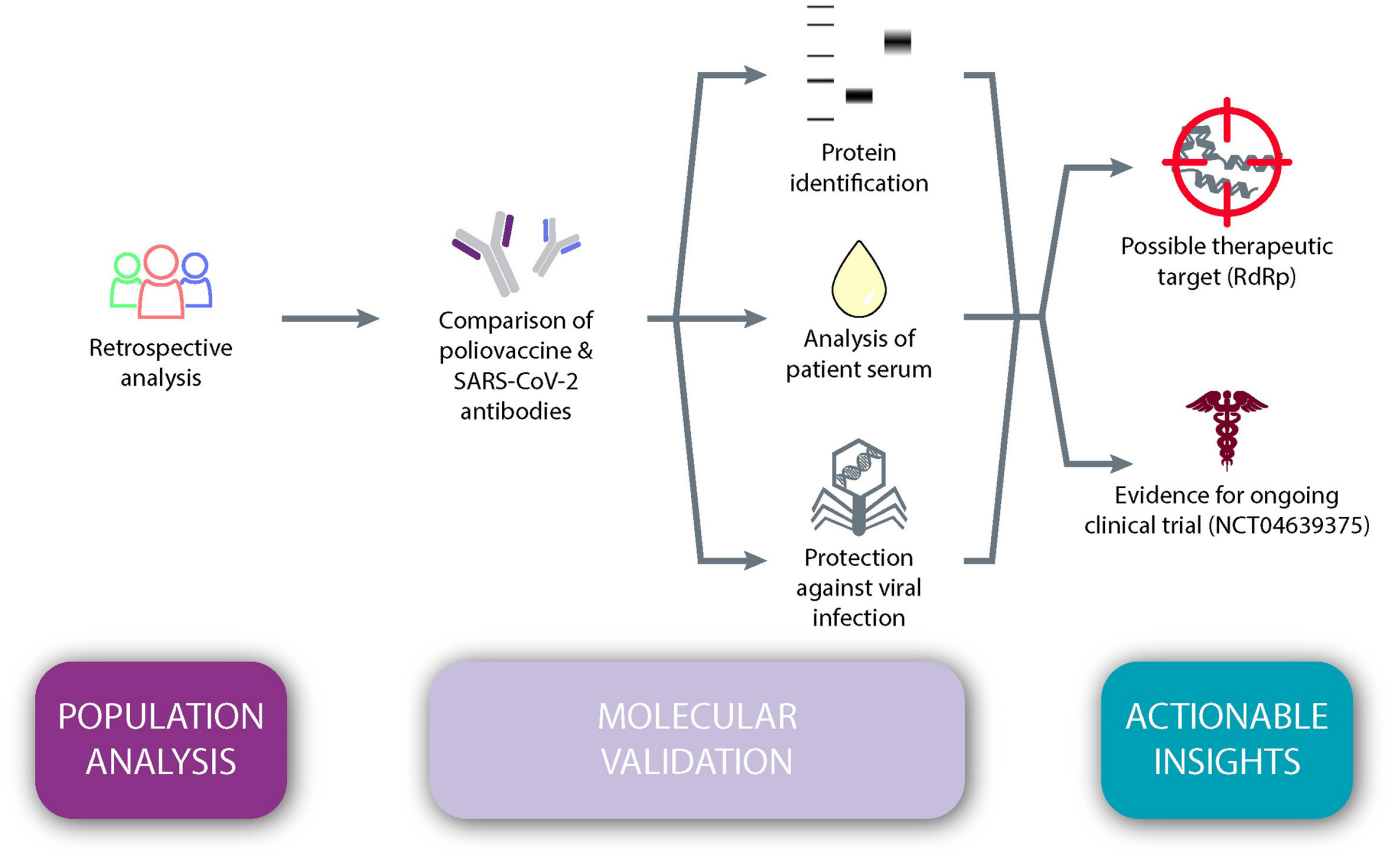
An observational study design testing the hypothesis that immunity against SARS-CoV-2 is dependent upon one's age and that poliovirus vaccination plays a vital role in this apparent protection.

The objective of the present study was to test the hypothesis that immunity against SARS-CoV-2 is dependent upon one's age, and that poliovirus vaccination plays a vital role in this apparent protection (Figure 1). We conducted a retrospective population analysis to assess whether there was an association between age and the prevalence and/or mortality of COVID-19 across the globe. Next, we evaluated whether antibodies induced by a childhood vaccine (IPV) may have similarities to SARS-CoV-2 antibodies. Retrospective analyses on sera from immunized individuals furthered this exploration through protein identification, as well as *in vitro* examinations of protein reactivity and inhibition of viral replication of SARS-CoV-2. Taken together, our results suggest actionable insights, namely a new possible therapeutic target, RdRp protein, and foundational evidence for a Phase IV clinical trial, which is currently underway in the United States.

## Methods

### Study Design

Here, we report two separate sets of analyses. First, we report a retrospective, observational statistical population analysis of COVID-19 prevalence and mortality with respect to age. Second, we perform a retrospective analysis on sera collected from 204 individuals to explore the molecular basis for similarities between poliovirus and SARS-CoV-2.

Written informed consent was obtained from all participants. Among those who had recently been inoculated with IPV, adults had been immunized with a 0.5 mL booster shot (standard dosage in the United States) and children had been vaccinated in four stages according to age-defined childhood vaccination protocols, set by the Centers for Disease Control and Prevention (CDC). IRB approval for the collection of adult sera was obtained from BioMed IRB, and pediatric samples were purchased from Discovery Life Sciences (Huntsville, AL) in accordance with the approved IRB protocol Alabama Biobank Research Trial 001 V3 (ABRT 001-V3).

### Relationship Between Median Age and COVID-19 Prevalence and Mortality

In order to evaluate if there was a relationship between countries' median age and prevalence or mortality of COVID-19, data on the total number of cases and deaths from the Johns Hopkins University Coronavirus Resource Center were compiled for the 100 countries with the highest prevalence of SARS-CoV-2 (13). Prevalence and death rates were then calculated for each country based on total population size, as of February 10, 2021. Median age for each country was then included for correlation analyses, calculated using the “corrplot” package from R Studio software, version 1.4.1103 (https://cran.r-project.org/index.html), between median age and SARS-CoV-2 prevalence and death rates.

### Cloning and Expression of SARS-CoV-2 RdRp

The full length *nsp12* gene was cloned into an AmeriDx® *E. coli* over-expression vector pADX75. Similarly, the poliovirus RdRp polyoprotein region 5286bp was amplified to 6564bp (NCBI accession number ALP31139) and cloned into the pADX75 vector. These two vectors were transformed into the BL21 strain respectively. The BL21 strains harboring the vectors were selected and used for protein expression and purification, according to standard protein purification protocols.

### Enzyme-Linked Immunosorbent Assay

An enzyme-linked immunosorbent assay (ELISA) was used for a quantitative analysis of the virus antibody in human sera. ELISA tests were carried out according to standard lab operating procedures. Briefly, the poliovirus vaccine (IPV)'s RdRp full length protein and the SARS-CoV-2 RdRp antigen were coated on the ELISA plate, respectively, and the human serum harboring the primary antibody was applied to each well. The primary antibody bound to the antigen coated on the well, and then the horse radish peroxidase (HRP) conjugated goat anti-human IgG/A/M secondary antibody, which recognizes the primary human antibody, formed a complex with the antigen, antigen-antibody-secondary antibody-HRP. TMB substrate was then added to the ELISA plate. Once the HRP enzyme turned the solution color to blue, the reaction was stopped by adding 2 M sulfuric acid, which converted the solution color to yellow. An ELISA reader measured and recorded the activity. We performed non-linear regression on the raw data using a four-parameter fitting algorithm using the R package “drc,” (14) which resulted in relative serum response units (reported as “arbitrary units”).

### Western Blot

Recombinant RdRp proteins were separated by 4–20% SDS-PAGE (Sodium Dodecyl Sulfate Polyacrylamide Gel Electrophoresis), and then were transferred to the PVDF membrane. Mouse monoclonal antibody 4E6 was added as 1:1000 in Western blot buffer TBST, and the antibody bound to antigens on the membrane. This binding was captured using a HRP conjugated goat anti-mouse IgG/A/M secondary antibody that conjugated with HRP. The HRP's chemiluminescence substrate then displayed a change of color for the specific RdRp protein band.

### RdRp Polymerase Assay

RdRp activity was determined *in vitro* by a fluorescence method, which was modified from a technique developed by others for Zika virus RdRp (15). There was a sample vial and a control vial for each sample, where water replaced ribonucleoside-tri-phosphates (rNTPs). The enzymatic reaction consisted of 0.2 M Tris-HCl, pH 8.0, 0.125 M NaCl, 40 mM MgCl_2_, 10 mM spermidine-(HCl)_3_, 2.5 uM rNTPs, 2.5 mM DTT, and 40 nM Syto-82 fluorescence dye. The sample serum was diluted to 1,800 times with 1xPBS, and then 2 uL were added to both vials. Samples were incubated at 37°C for 30 min. The newly formed double stranded RNA then bound to the Syto-82 fluorescence dye, and emitted 589 nm light that could be recorded by a GeneScan^TM^ fluorescence reader. The fluorescence signal in each vial was quantified using the mean gray value in an ImageJ program (https://imagej.nih.gov/ij/index.html) (16). The fluorescence intensity difference between these two vials was designed to reflect the RdRp's relative activity, such that a positive value represented up-regulated enzymatic activity (normal replication, causing an individual to become ill), and a negative value represented down-regulated enzymatic activity (replication inhibited, preventing viral effects).

### Antiviral Assays

A cytopathic effect (CPE)-based antiviral assay was performed *in vitro* by infecting Vero-E6-cells in the presence or absence of immune sera to evaluate antiviral activity against SARS-CoV-2 (MEX-BC2/2020) (17). Most of the pediatric serum samples were <1 mL each, and thus not enough for a single CPE test assay, so we pooled samples from subjects of the same age. Vero E6 cells were seeded and incubated for 24 h. Sera were either pre-incubated first with target cells for 1 h at 37°C before infection with SARS-CoV-2, or cells were treated with SARS-CoV-2 for 3 h at 37°C to allow viral adsorption, and then the serum specimens were added to the target cells without removing the viral inoculum. The cells were challenged with the viral inoculum and re-suspended in DMEM with 2% FBS (DMEM2). The test sera and virus were maintained in the cell culture media for 96 h. As a surrogate marker for inhibition of viral replication, inhibition of SARS-CoV-2-induced CPE was measured using uninfected Vero E6 cells as mock control, and the cells infected with SARS-CoV-2 virus only (no serum) as vehicle control. Cell viability was monitored with the neutral red (NR) uptake assay. The average absorbance at 540 nm (A540) from each well on a 96-well plate was calculated in percentage using the following formula:

$$\begin{array}{l} \text{A540}=\frac{\text{Average OD540 of the testing well }-\text{ OD540 of vehicle}}{\text{OD540 of Mock }-\text{ OD540 of Vehicle}} \end{array}$$

where vehicle is the O.D. from virus-infected cells without any testing serum samples, and mock is the O.D. value from the uninfected cells.

## Results

### Relationship Between Median Age and Prevalence and/or Mortality Aspects of COVID-19

**Figure 2:**
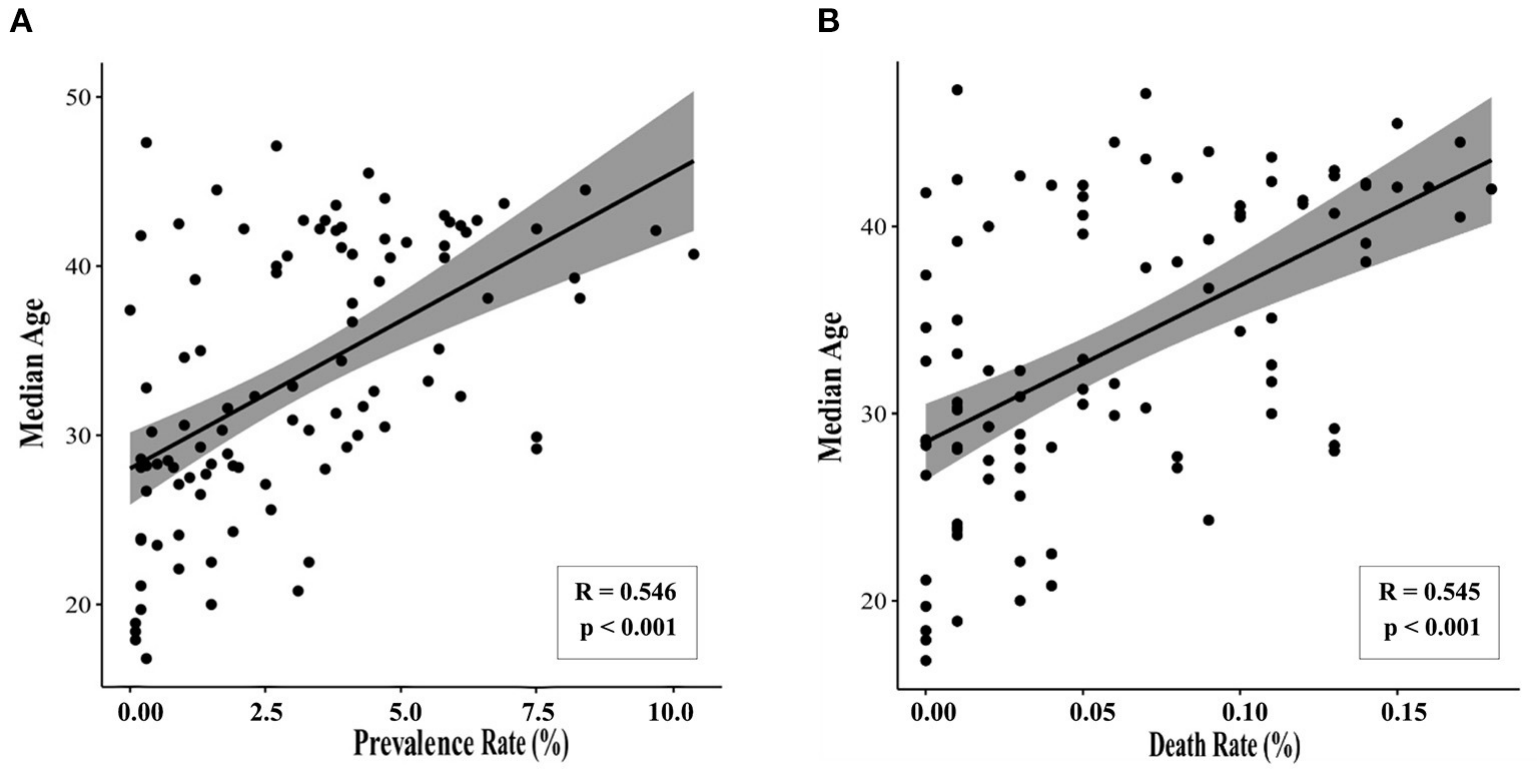
Median age correlates with SARS-CoV-2 prevalence and death rate. Data were collected for the 100 countries with the highest prevalence of SARS-CoV-2 (accessed February 10, 2021). Statistically significant correlations were observed between median age and **(A)** prevalence of SARS-CoV-2 (Pearson *r* = 0.546, *p* < 0.001) and **(B)** COVID-19-related death rate (Pearson *r* = 0.545, *p* < 0.001).

We first sought to determine whether there are age-related differences in COVID-19 prevalence and mortality by performing a retrospective literature study. Using data from the 100 countries with the highest rates of SARS-CoV-2, correlation analyses show there is a positive association between a country's median age and prevalence rate of SARS-CoV-2 (Pearson *r* = 0.546, *p* < 0.001). There is also a positive correlation between median age and the SARS-CoV-2-related death rate (Pearson *r* = 0.545, *p* < 0.001) (Figure 2). Countries with older national median ages were significantly more likely to have a higher prevalence of SARS-CoV-2, as well as a higher SARS-CoV-2-related death rate. While such an analysis has limitations, this result nevertheless suggests that there is indeed a statistically significant positive association between age and COVID-19 prevalence and mortality.

### Poliovirus RdRp Shows Antibody Overlap With SARS-CoV-2 RdRp

**Figure 3:**
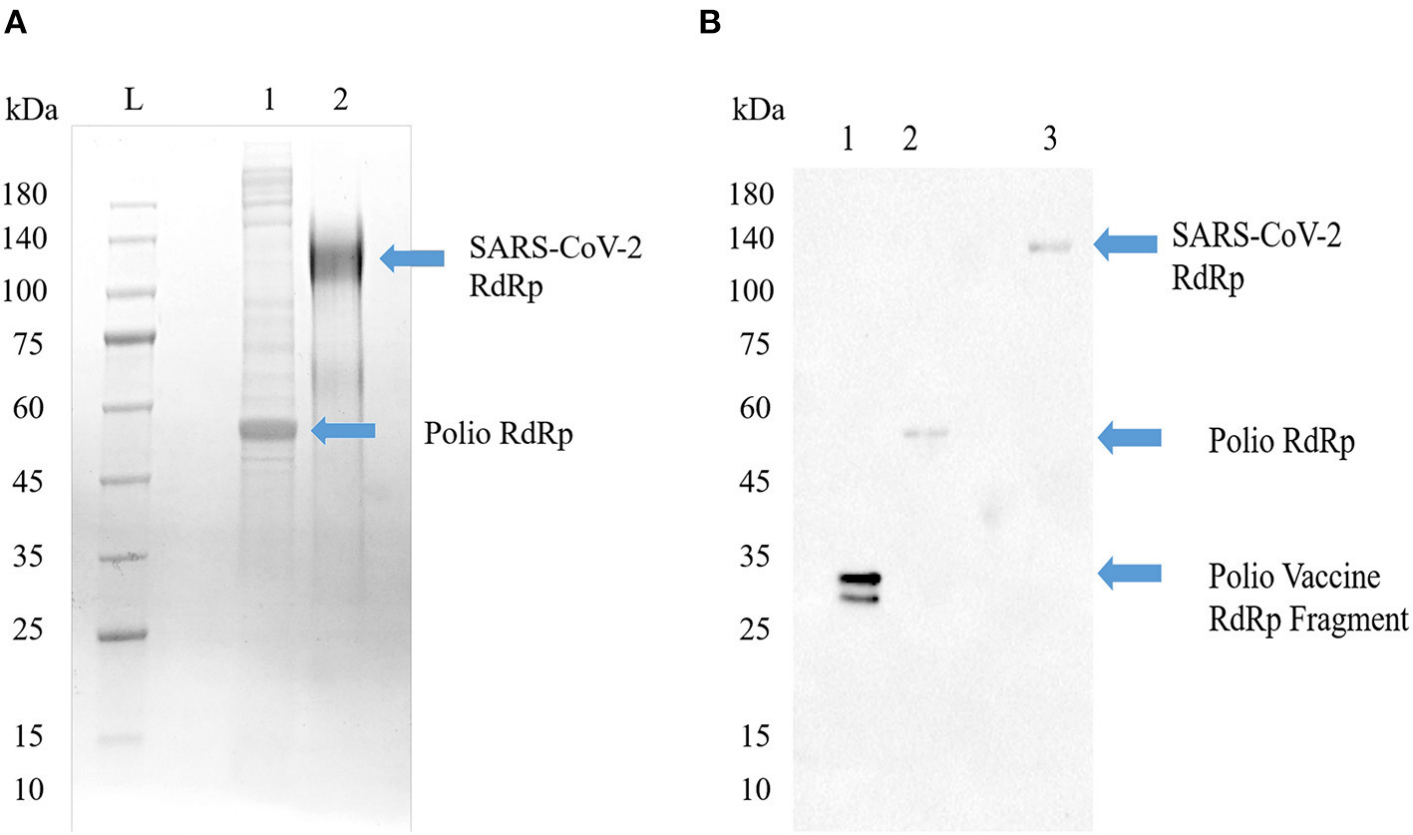
Poliovirus RdRp contains one or more epitopes recognized by SARS-CoV-2 RdRp antibodies. **(A)** SDS-PAGE image of Poliovirus RdRp proteins from bacterial cells (Lane 1) and full SARS-CoV-2 RdRp (Lane 2). The poliovirus RdRp from bacterial cells seems to be degraded or not post-translationally modified. Both viral forms of the RdRp in their native configuration have been reported to have a molecular weight around 130 kD. **(B)** Western blot identification of the antigenic activity of RdRp proteins. The primary antibody was a mouse monoclonal antibody to SARS-CoV-2 RdRp protein, and the secondary antibody was a goat anti-mouse IgG/A/M HRP. Lane 1, a vaccine sample shows a line at about 35 kD; Lane 2, poliovirus RdRp expressed in bacterial cells shows a molecular weight around 50–55 kD, representing an un-modified and possibly truncated form of the RdRp; Lane 3, SARS-CoV-2 RdRp, shows a line around 130 kD.

Having shown that COVID-19 prevalence and mortality is age dependent, we next sought to determine what role—if any—previous inoculation with poliovirus may play. Thus, we explored whether antibodies generated by SARS-CoV-2 RdRp protein could recognize recombinant poliovirus RdRp antigens. The tertiary and quaternary structures of both the RdRp from poliovirus and SARS-CoV-1 are right-handed palm type (11), and SARS-CoV-1 and SARS-CoV-2 have similar structures (18). As shown in Figure 3, both RdRp can be recognized by SARS-CoV-2 RdRp monoclonal antibody 4E6.

### Antibody Titers Before and After Poliovirus Vaccination Show Conferred SARS-CoV-2 Immunity

**Figure 4:**
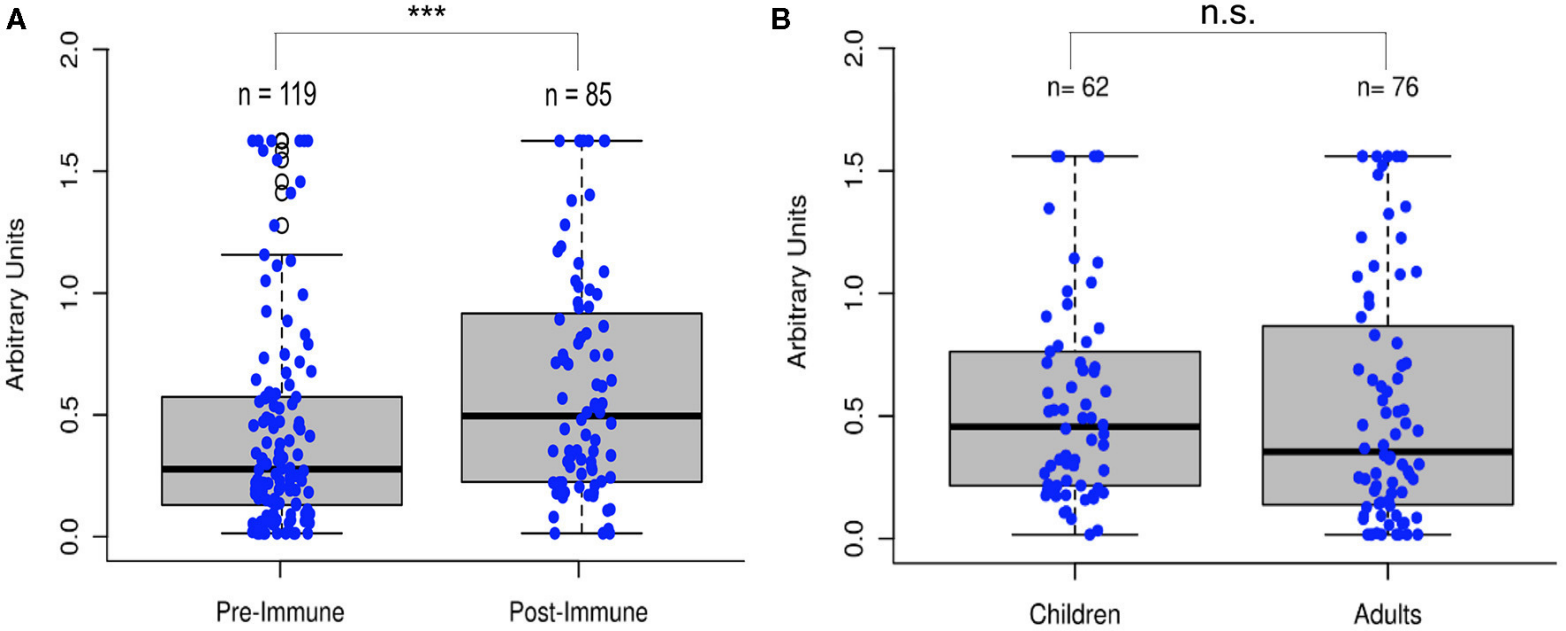
Poliovirus vaccination shows significant increase in RdRp reactivity. **(A)** Comparison of sera (*N* = 204) before and after poliovirus immunization showed that adults gained significantly more antibodies following the poliovirus vaccination (Two-tailed *t*-test, ***p < 0.001). **(B)** Comparison of pediatric and immunized adult sera showed no significant differences between the two groups (Two-tailed *t*-test, *p* = 0.85), with immunized adults displaying similar antibody responses as immunized children.

To test the hypothesis that poliovirus vaccination induces an immunity to SARS-CoV-2, we analyzed 204 serum samples from 119 individuals who had not recently been vaccinated with IPV, referred to as “pre-immunized samples,” and 85 individuals who had recently received IPV, referred to as “post-immunized individuals,” for anti-RdRp antibody titers. While the immune response to antigenic RdRp protein varies from person to person, sera from those who had been vaccinated for poliovirus showed a significant increase in antibody titers (Two-tailed *t*-test, *p* < 0.001, Figure 4A). Additionally, we compared a late-stage immunized pediatric group (children who had received at least three of the four CDC recommended polio dosages, depending on their age, *N* = 62, age 1–10) to a recently immunized adult group (*N* = 76 of the total 85 post-immunized samples, age 25–89) and observed no significant difference in antigenic specificity between pediatric and re-immunized adult sera (Two-tailed *t*-test, *p* = 0.85), indicating immunized adults display similar antibody responses as children that have received their childhood vaccinations (Figure 4B).

### Antiviral and Enzymatic Activity of Polio-Immune Sera

**Figure 5:**
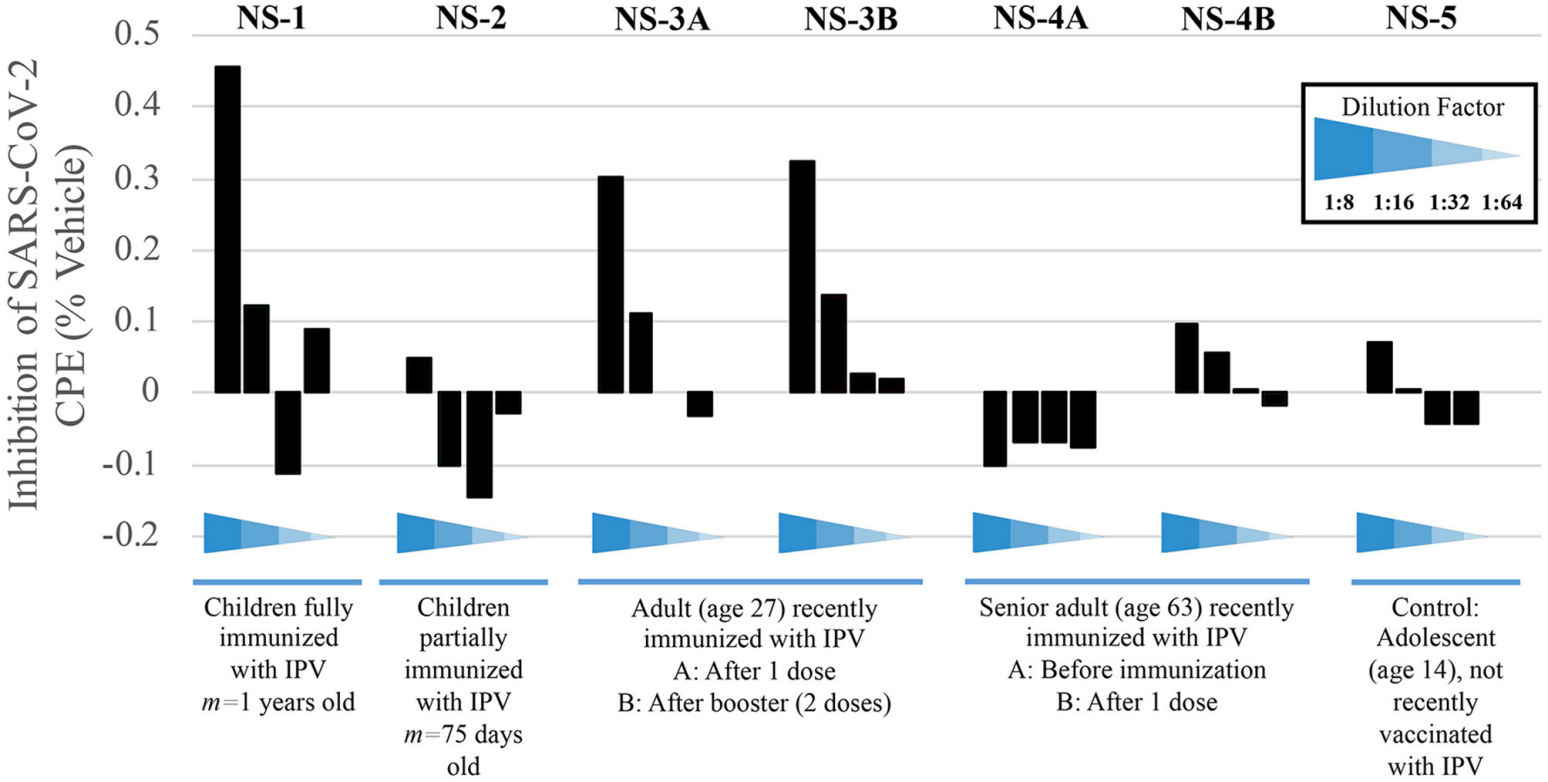
Sera from immunized individuals demonstrate higher inhibition of SARS-CoV-2-induced CPE. Sera from five test subjects of varying ages and IPV-immunization stages were added before viral adsorption. Values show the inhibition of the SARS-CoV-2-induced CPE, as a surrogate marker for the inhibition of viral replication; inhibition percentage was calculated by average O.D. 540 nm from each well, normalized with mock and vehicle control from the same plate (see Methods).

Having observed that the poliovirus vaccine significantly affects SARS-CoV-2 RdRp reactivity, our next goal was to understand the mechanism of action behind this phenomenon. Inhibition of SARS-CoV-2-induced cytopathic effect (CPE) in Vero-cell culture was used as a surrogate marker for the inhibition of viral replication. Polio-immune serum demonstrated an antiviral effect in Vero cells at dilutions 1:8 to 1:32, which was stronger when the antisera were pre-incubated with cells, rather than added after viral adsorption. Samples from children fully immunized with IPV (NS-1) and young adults (NS-3A and NS-3B) show the strongest inhibition of viral CPE when added to cell culture prior to virus (Figure 5). The sample labeled NS-1 was from pooled sera from children who were on average 1 years old, collected prior to the onset of the SARS-CoV-2 pandemic; these children would have been immunized against polio as part of national immunization campaigns. NS-2 samples were pooled from infants who were on average 75 days old; these infants would not have been fully immunized against polio. The remaining samples depicted in Figure 5 were randomly selected from the adult pool of paired samples to demonstrate differences in immunity, based on age and IPV immunization. Sample NS-3A is from a 27-year-old subject, who was recently given IPV. The NS-3B sample is from the same subject after obtaining a second IPV booster. Sample NS-4A is serum from a 63-year-old female subject pre-IPV vaccination; NS-4B is her post-vaccination serum sample. The NS-5 sample represents serum from a 14-year-old subject, who had not recently been vaccinated with an IPV booster.

Pediatric serum samples were pooled in order to perform the analysis for infants, as shown in Figure 5 (NS-1 and NS-2). While a lack of individual samples precludes statistical analysis, we are able to observe a qualitative trend that children who have received their childhood vaccinations by 1 year of age display stronger immunity to SARS-CoV-2, as evidenced by CPE levels, compared to children that are <4 months old and still very early in immunization protocols (NS-1 and NS-2, respectively). This result is expected if, as we propose, poliovirus immunization plays a role in the relative resistance to COVID-19 in younger populations. Samples NS-3A and NS-3B further demonstrate how polio immunization (a single inoculation, followed by a second booster 2 months later, respectively) inhibits SARS-CoV-2-induced CPE.

Protection from poliovirus or SARS-CoV-2 declines as one ages. No inhibition of SARS-CoV-2-induced CPE is evident pre-vaccination (NS-4A), though older adults' immune systems can respond to an IPV booster (NS-4B). Here, data show approximately a 35% increase of protection from viral CPE, relative to pre-vaccination levels. While the immunity is lower in the 63-year-old adult, compared to the 27-year-old adult, IPV provides some protection that the teenager (NS-5), who was not recently vaccinated, does not have.

### Inhibition of RdRp Activity Is Inhibited *in vitro* by Sera From Immunized Individuals

**Figure 6:**
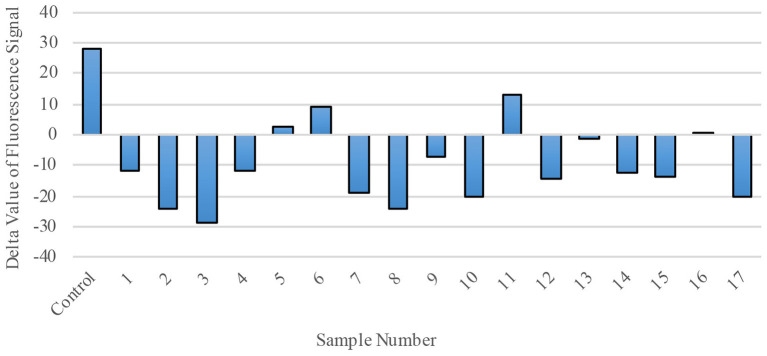
RdRp Enzymatic Activity is blocked by polio-immune serum. RdRp polymerase activity was evaluated by fluorescence staining of *de novo* synthesized RNA. In the presence of diluted serum (1:1800), RdRp's activity is inhibited. Each bar represents one serum sample (17 randomly-selected samples compared to a control).

Finally, we explored RdRp as a possible therapeutic target by evaluating the effect of polio-immune serum on SARS-CoV-2 RdRp activity in living cells. Randomly-selected sera from 17 different polio-immunized subjects (seven children; 10 adults) were added to the RdRp reaction mix at a dilution of 1:1800. RdRp activity was then measured by fluorescence signal (Figure 6), with a positive value representing regular enzymatic RdRp activity (virus will replicate normally and cause the individual to become ill) and a negative value representing inhibition of the RdRp enzyme (sera from immunized individuals reduce the possibility of viral replication and inhibit the virus from entering the cell). Of the 17 randomly-selected sera from immunized individuals, 13 (76.5%) were effective in inhibiting the enzymatic activity of RdRp.

## Discussion

With the continued and global spread of SARS-CoV-2, there are open questions regarding the virus' interaction with the immune system, particularly across various age groups. The interplay between SARS-CoV-2 and other vaccines, such as the poliovirus vaccine, may offer insights into potential therapeutics against COVID-19. Here, we performed a retrospective analysis on sera from 204 individuals to explore the role of poliovirus vaccination in ameliorating the impact of COVID-19 in a population. Our results show poliovirus vaccination raises antibodies that cross-react with SARS-CoV-2, with the primary target of these antibodies being the RdRp of poliovirus and coronavirus. Sera from individuals immunized against poliovirus, when added to cell cultures before and after viral infection, provide protection from viral CPE. When antisera are added to the RdRp replication system, RNA replication is reduced. Taken together, these results have two important implications.

First, an IPV-induced adaptive humoral immune response suggests that poliovirus immunization in infants and children as part of national vaccination efforts provides some level of protection from SARS-CoV-2 infection until young adulthood. The association between national median age and COVID-19 prevalence and mortality rates across countries suggests a lack of immunity to SARS-CoV-2 in older adults, compared to younger individuals, who may still possess immunity from childhood vaccinations, including poliovirus inoculations. Similar demographic patterns were observed from infection prevalence and mortality rates during the 2003 SARS-CoV epidemic (4), where children were less likely to exhibit symptoms related to SARS, compared to older adults (19). Likewise, such relationships have been evident during the COVID-19 pandemic, as the mortality for people aged 0–24 is substantially less than the mortality in people older than 25 (20–22). Childhood vaccinations may have raised antibodies to SARS-CoV-2 in younger individuals, reducing both the prevalence and mortality of COVID-19. Other studies have suggested vaccines that induce an innate immune response, such as BCG, MMR, and poliovirus, may be useful in preventing SARS-CoV-2 (7, 23).

Second, we propose that these results help explain why there is an apparent age-dependent outcome following SARS-CoV-2 infection (1–3). There are many exceptions to this observation, as disease is seen in these younger age groups, but at a much lower incidence than the life-threatening disease often seen in adults (24, 25). While younger individuals may still possess immunity from childhood vaccinations, older adults can mount an adaptive immune response to SARS-CoV-2 following poliovirus re-immunization. Without IPV or OPV boosters, older adults may remain at higher risk of severe disease and mortality (2, 26, 27). This retrospective study provides a preliminary understanding of the role poliovirus vaccination has in inhibiting viral replication of SARS-CoV-2. We are currently conducting a larger clinical trial (NCT04639375) to provide deeper validation of the potential utility of the safe and effective poliovirus vaccine as a prophylactic measure against COVID-19 infection (28).

Due to its observational nature, the population analytics aspect of this study is subject to potential biases, including confounding, selection bias, and measurement bias. For instance, variation in risk communication, social restrictions, access to protective equipment, availability of other resources, and public adherence to risk mitigation measures may have contributed to the extent to which COVID-19 impacted certain countries. Other potential confounders may include unobserved factors, such as socioeconomic status and travel history (29). The statistical analysis linking median age and the prevalence of COVID-19 symptoms fails to account for many variables, such as any possible co-morbidities that may exacerbate COVID-19. Additionally, the prevalence and recorded death rates are dependent upon many factors that introduce variability and potential error, such as the accuracy and credibility of reported COVID-19 cases. Deaths related to COVID-19 may be underreported if co-morbidities were listed as the main cause of death, or if COVID-19 was not diagnosed (30). Prevalence of COVID-19 may also be underreported due to asymptomatic cases that may not have been tested for COVID-19. While these challenges limit the interpretation of the present results, our analysis nevertheless suggests the need for more detailed studies with larger cohorts that specifically address some of these variables. Further, the sample sizes reported as part of our retrospective sera analysis were small due to the scope of our pilot study, but require larger numbers to adequately compute statistics for deeper conclusions.

The poliovirus vaccine poses minimal risk. The World Health Organization recommends poliovirus vaccine boosters for adults traveling to high-risk zones of polio infection (31). In comparison to other vaccines that are currently being tested for COVID-19 prevention, poliovirus vaccines, both IPV and OPV, are readily available with prior pharmacological, toxicity, chemical, manufacturing, and control data. Possible risks have been studied for over 60 years and all processes have been well-documented and established. Given the current climate of vaccine hesitancy with the COVID-19 vaccine roll-out, and projected vaccine shortage across the globe, the results reported here suggest more attention should be given to a vaccine that 90% of the world's population received as children. While these data show proteins other than “spike” proteins, such as RdRp, may be suitable targets for immunity and vaccine development, IPV may also be used to complement the new Emergency Use Authorization (EUA) COVID-19 vaccines, to further boost immunity and enhance the population's health. The results described here could be strengthened by additional studies performed in multiple countries—including low-income countries—to explore any possible variation in CPE of sera, especially among countries that administer OPV, compared to those that utilize IPV for childhood vaccinations.

This retrospective serological study demonstrates poliovirus vaccination produces antibodies that inhibit RdRp function, thereby preventing viral replication that may cause disease progression in infected individuals. Based on these laboratory findings, we conclude polio-vaccinated individuals (children or adults who have recently been immunized with IPV) may have a high level of protection against COVID-19 that non-inoculated individuals do not have.

## Data Availability Statement

The original contributions generated for this study are included in the article/supplementary material, further inquiries can be directed to the corresponding author/s.

## Ethics Statement

The studies involving human participants were reviewed and approved by BioMed IRB for the collection of adult sera, and pediatric samples were purchased from Discovery Life Sciences (Huntsville, AL) in accordance with the approved IRB protocol Alabama Biobank Research Trial 001 V3 (ABRT 001-V3). Written informed consent to participate in this study was provided by the participants' legal guardian/next of kin.

## Author Contributions

QX conceptualized the study. LE and RC provided supervision. YJ, QL, and LJ performed experimental analyses. YJ performed statistical analyses. BC and YJ performed population analytics. BC, YJ, JA, JY, and QX wrote and prepared the manuscript. All authors edited and approved the final manuscript.

## Conflict of Interest

BC did not receive any personal fees from E-Mo Biology, Inc. while writing this manuscript, though is pending negotiations for future interests. JA and RC report personal fees from E-Mo Biology, Inc. YJ reports personal fees from GeneScan Diagnostics, LLC. QX holds an executive position in and reports personal fees from E-Mo Biology, Inc. E-Mo Biology, Inc. has a patent pending. The remaining authors declare that the research was conducted in the absence of any commercial or financial relationships that could be construed as a potential conflict of interest.

## Publisher's Note

All claims expressed in this article are solely those of the authors and do not necessarily represent those of their affiliated organizations, or those of the publisher, the editors and the reviewers. Any product that may be evaluated in this article, or claim that may be made by its manufacturer, is not guaranteed or endorsed by the publisher.
